# Supplementary material for: Accuracy of prenatal screening for congenital heart disease in population: A retrospective study in Southern France
Source: PLoS One. 2020 Oct 5;15(10):e0239476. doi: 10.1371/journal.pone.0239476 (PMC7535055; doi:10.1371/journal.pone.0239476)
Supplement: S1 File — VD: Right ventricle; VG: Left ventricle; OD: Right atrium; OG: left atrium; VP: Pulmonary vein; Ao: Aorta; AP: Pulmonary artery; VCS: superior vena cava. (PDF) [file pone.0239476.s001.pdf]

Transposition of the great arteries: the keys to screening

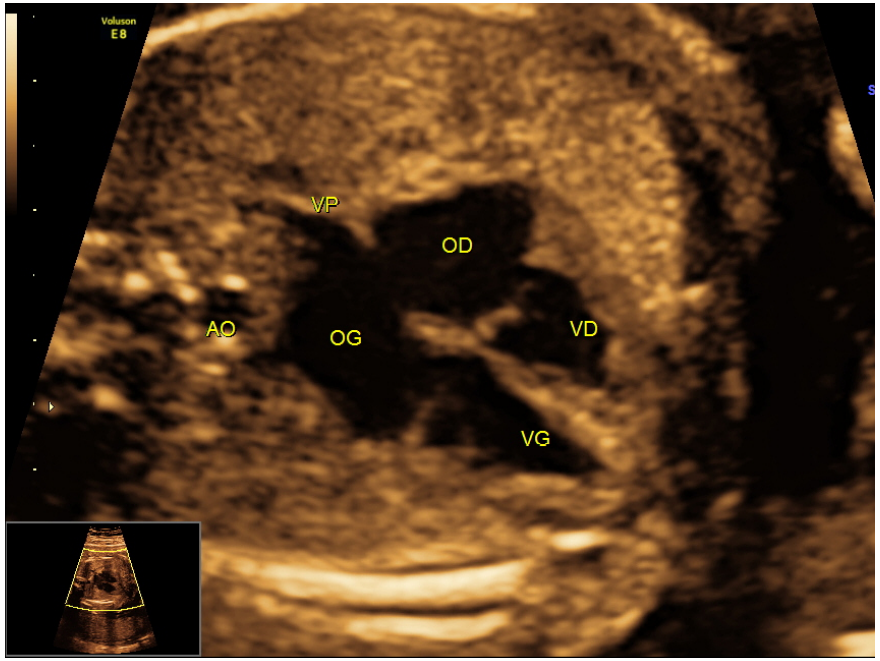

The cut of the 4 cavities is normal

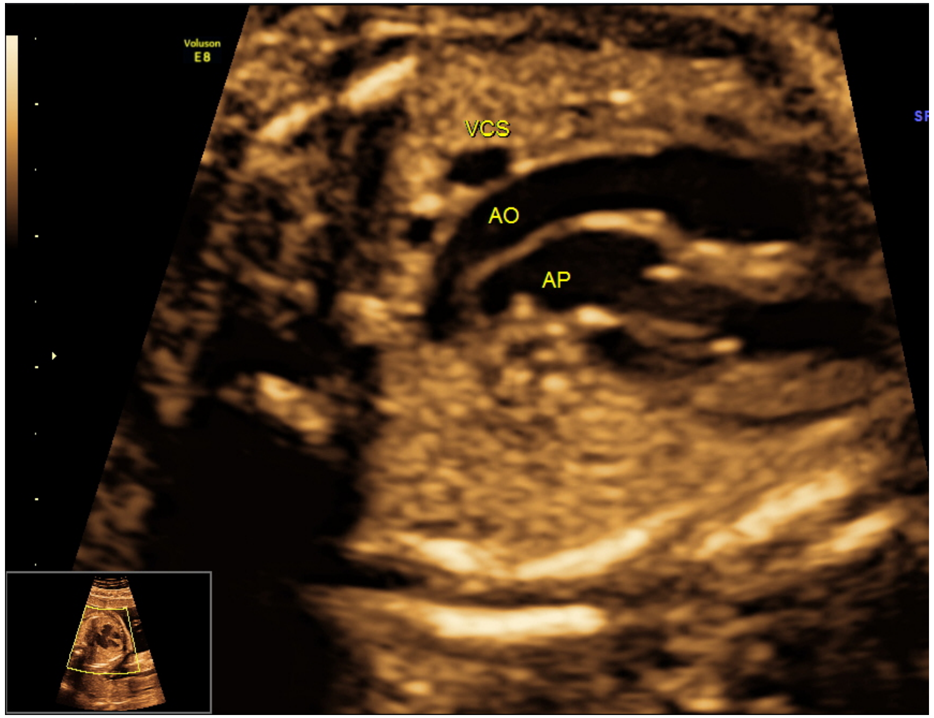

Parallel aspect of the great vessels in “gun barrels”

- The aorta comes out of the right ventricle
- The pulmonary artery comes out of the left ventricle
