# Supplementary material for: Accuracy of prenatal screening for congenital heart disease in population: A retrospective study in Southern France
Source: PLoS One. 2020 Oct 5;15(10):e0239476. doi: 10.1371/journal.pone.0239476 (PMC7535055; doi:10.1371/journal.pone.0239476)

Total anomalous pulmonary venous returns: the keys to screening

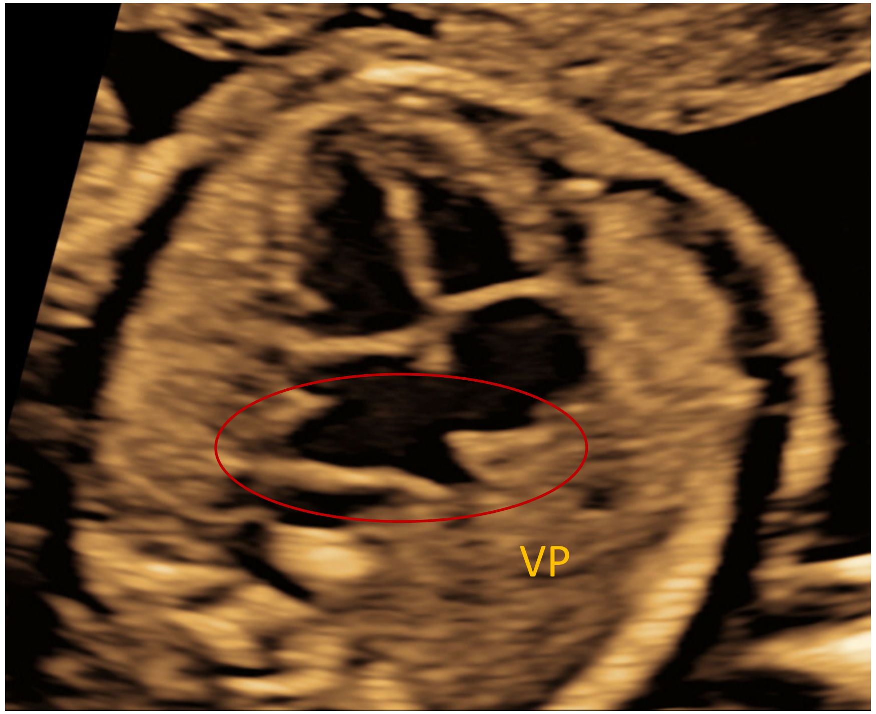

Lack of pulmonary veins in the left atrium

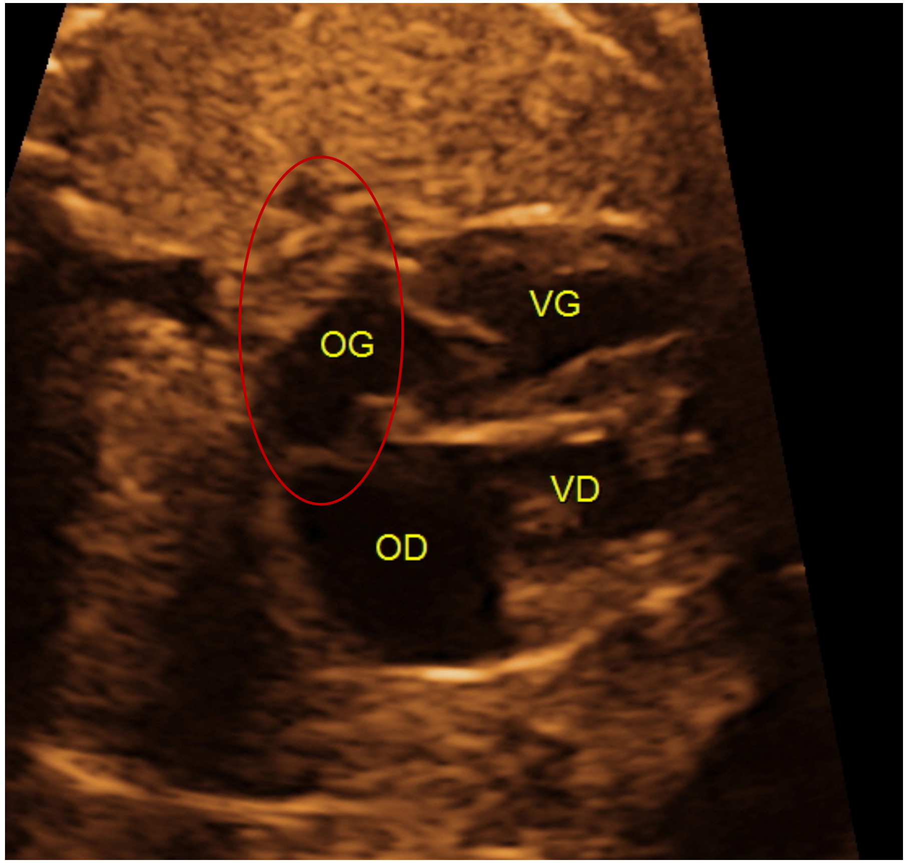

Supplement: S2 File — VD: Right ventricle; VG: Left ventricle; OD: Right atrium; OG: left atrium; VP: Pulmonary vein. (PDF) [file pone.0239476.s002.pdf]
