# Supplementary material for: Accuracy of prenatal screening for congenital heart disease in population: A retrospective study in Southern France
Source: PLoS One. 2020 Oct 5;15(10):e0239476. doi: 10.1371/journal.pone.0239476 (PMC7535055; doi:10.1371/journal.pone.0239476)
Supplement: S3 File — VD: Right ventricle; VG: Left ventricle; VP: Pulmonary vein; Ao: Aorta; AP: Pulmonary artery; VCS: superior vena cava. (PDF) [file pone.0239476.s003.pdf]

Aorta coarctation: the keys to screening

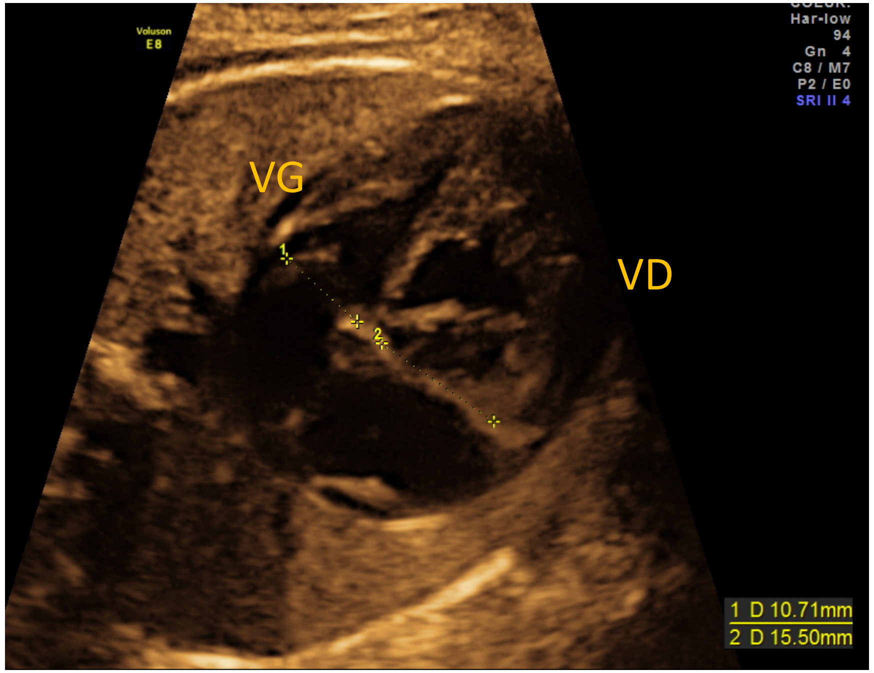

Asymmetry of right> left cavities

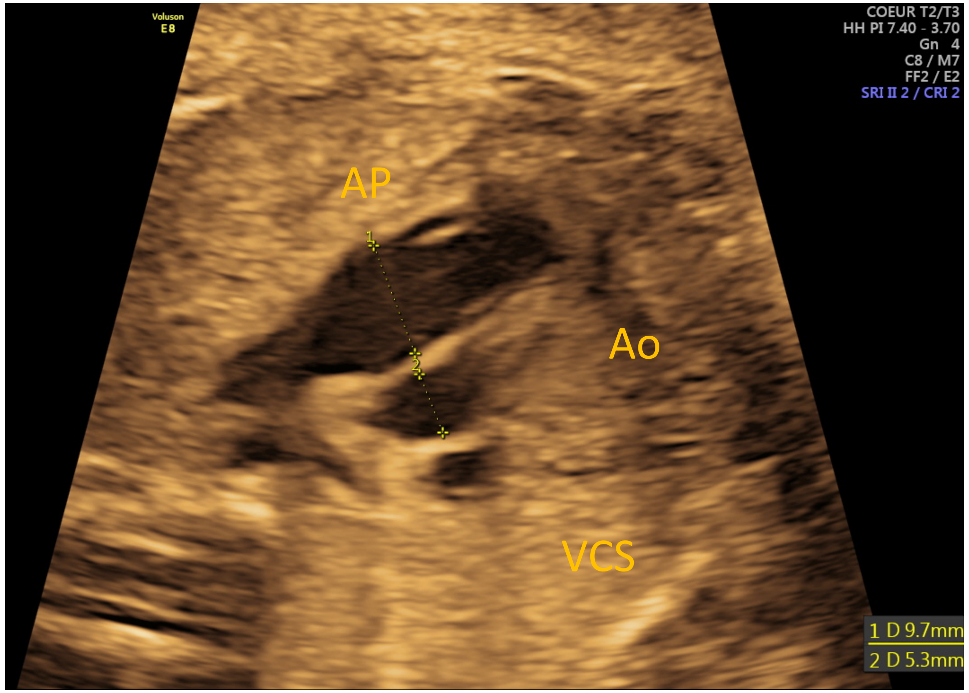

Significant risk of coarctation of the aorta if PA / Ao> 1.6
